# Deletion of mTORC1 Activity in CD4+ T Cells Is Associated with Lung Fibrosis and Increased γδ T Cells

**DOI:** 10.1371/journal.pone.0163288

**Published:** 2016-09-20

**Authors:** Christine L. Vigeland, Samuel L. Collins, Yee Chan-Li, Andrew H. Hughes, Min-Hee Oh, Jonathan D. Powell, Maureen R. Horton

**Affiliations:** 1 Department of Medicine, Johns Hopkins University School of Medicine, Baltimore, Maryland, United States of America; 2 Department of Oncology, Johns Hopkins University School of Medicine, Baltimore, Maryland, United States of America; Centre National de la Recherche Scientifique, FRANCE

## Abstract

Pulmonary fibrosis is a devastating, incurable disease in which chronic inflammation and dysregulated, excessive wound healing lead to progressive fibrosis, lung dysfunction, and ultimately death. Prior studies have implicated the cytokine IL-17A and Th17 cells in promoting the development of fibrosis. We hypothesized that loss of Th17 cells via CD4-specific deletion of mTORC1 activity would abrogate the development of bleomycin-induced pulmonary fibrosis. However, in actuality loss of Th17 cells led to increased mortality and fibrosis in response to bleomycin. We found that in the absence of Th17 cells, there was continued production of IL-17A by γδ T cells. These IL-17A+ γδ T cells were associated with increased lung neutrophils and M2 macrophages, accelerated development of fibrosis, and increased mortality. These data elucidate the critical role of IL-17A+ γδ T cells in promoting chronic inflammation and fibrosis, and reveal a novel therapeutic target for treatment of pulmonary fibrosis.

## Introduction

Pulmonary fibrosis is a rapidly progressive, fatal lung disease. The pathogenesis of this disease is not fully understood, but it is believed to develop as a result of dysregulated, excessive wound healing[1, 2]. Pro-inflammatory and pro-fibrotic cytokines such as TNF-α, IL-1β, and TGF-β, as well as M2 macrophages are believed to play a role in promoting this response[3, 4]. Currently, the only approved therapies slow the progression of disease, but do not arrest or reverse the fibrosis[5, 6]. Thus, there is an urgent need for improved understanding of the pathogenesis of pulmonary fibrosis so that novel therapies can be developed. Prior work in both animal models as well as humans has implicated IL-17A in driving the development of pulmonary fibrosis[7–9]. In animal models, IL-17A is produced by Th17 cells and γδ T cells within the lungs after fibrotic stimuli[7, 10, 11]. However, the contributions of Th17 cells and γδ T cells to the development of pulmonary fibrosis remains unclear. While Th17 cells have been implicated in promoting the development of fibrosis through production of IL-17A, γδ T cells have been found to ameliorate lung fibrosis[7, 10, 12]. This effect of γδ T cells has been hypothesized to be mediated via production of IL-17A, CXCL10, IL-22, or IFN-γ[10, 13–15].

It has been previously shown that mTOR is a critical regulator of CD4+ T cell differentiation[16]. mTOR signals through two signaling pathways, mTORC1 and mTORC2. Loss of mTORC1 signaling prevents the differentiation of naïve CD4+ T cells into Th1 and Th17 cells, while loss of mTORC2 signaling prevents differentiation into Th2 cells[16]. The protein raptor is necessary for mTORC1 signaling, but not mTORC2 signaling. Thus, selective deletion of raptor linked to expression of CD4 creates CD4+ T cells that are unable to differentiate into a Th1 or Th17 phenotype.

Previous work investigating the role of IL-17A-producing cells in pulmonary fibrosis has utilized the intratracheal bleomycin model. This model causes acute inflammation that progresses to chronic inflammation and fibrosis over 14 to 21 days[17]. These phases of injury have been shown to have different mechanisms of regulation[18]. Thus, it is important to differentiate between the acute and chronic inflammatory phases. Within human disease, however, there is often not a recognized acute inflammatory phase[19]. Fibrosis gradually develops over years. Thus, in order to mimic human pathology more closely, we employed a model involving repeated intraperitoneal injections of bleomycin given over a period of four weeks to induce pulmonary fibrosis with less acute inflammation[12]. This protocol produces pulmonary fibrosis by day 42 that continues to progress through day 70[12]. By utilizing this model, we can focus our investigation on the chronic inflammation and progressive fibrosis that is more characteristic of human disease.

We hypothesized that, because they are unable to generate Th17 cells, our CD4 raptor knockout mice would have attenuated injury from bleomycin. Surprisingly, in the CD4 raptor knockout mice, in the absence of Th17 cells, we observed increased mortality and fibrosis. Further investigation revealed robust IL-17A production by γδ T cells in the lungs. Our data reveals the critical role of γδ T cells in promoting the development of fibrosis.

## Results

### Loss of CD4 raptor expression causes increased mortality and fibrosis in response to bleomycin

**Fig 1:**
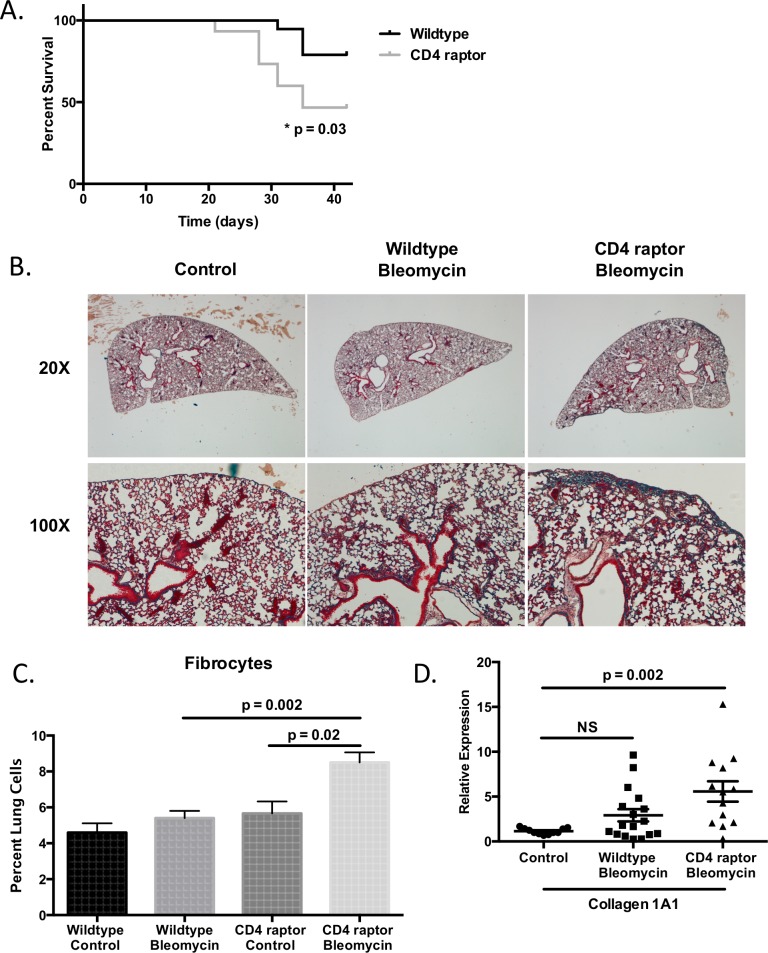
CD4 raptor knockout mice develop increased fibrosis and mortality in response to bleomycin. (A) Survival curves for wild type and CD4 raptor knockout mice following i.p. bleomycin. Data from 3 pooled experiments, n = 15–19 mice per group. *P* = 0.03 by Log-rank test. (B) Masson’s trichrome staining of lungs on day 42 following i.p. bleomycin at x20 (upper panels) and x100 (lower panels) magnification. (C) Lung fibrocytes on day 21 following i.p. bleomycin. Data shown from one representative experiment with a total of three replicates and n = 3–6 per group. (D) Expression of collagen 1A1 within the lungs on day 21 following i.p. bleomycin. Data shown from three pooled experiments with n = 11–17 per group. (C-D) Error bars represent one standard error. Significance determined by one-way ANOVA followed by Sidak’s multiple comparison’s test.

To induce pulmonary fibrosis, wild type and CD4 raptor knockout mice were given intraperitoneal (i.p.) injections of bleomycin over 28 days and harvested at day 42. Prior work by our group has shown that this protocol induces pulmonary fibrosis by day 42 with little mortality[12]. However, in response to bleomycin, the CD4 raptor knockout mice had significantly increased mortality (Fig 1A). They developed fibrosis as shown by Masson-Trichrome staining, and developed it earlier in the course of treatment (Fig 1B). By day 21, just halfway through the protocol, the CD4 raptor mice had evidence of fibrosis with an increase in the number of lung fibrocytes and expression of collagen 1A1 within the lungs (Fig 1C and 1D).

### CD4 raptor knockout mice have increased γδ T cells within the lungs

**Fig 2:**
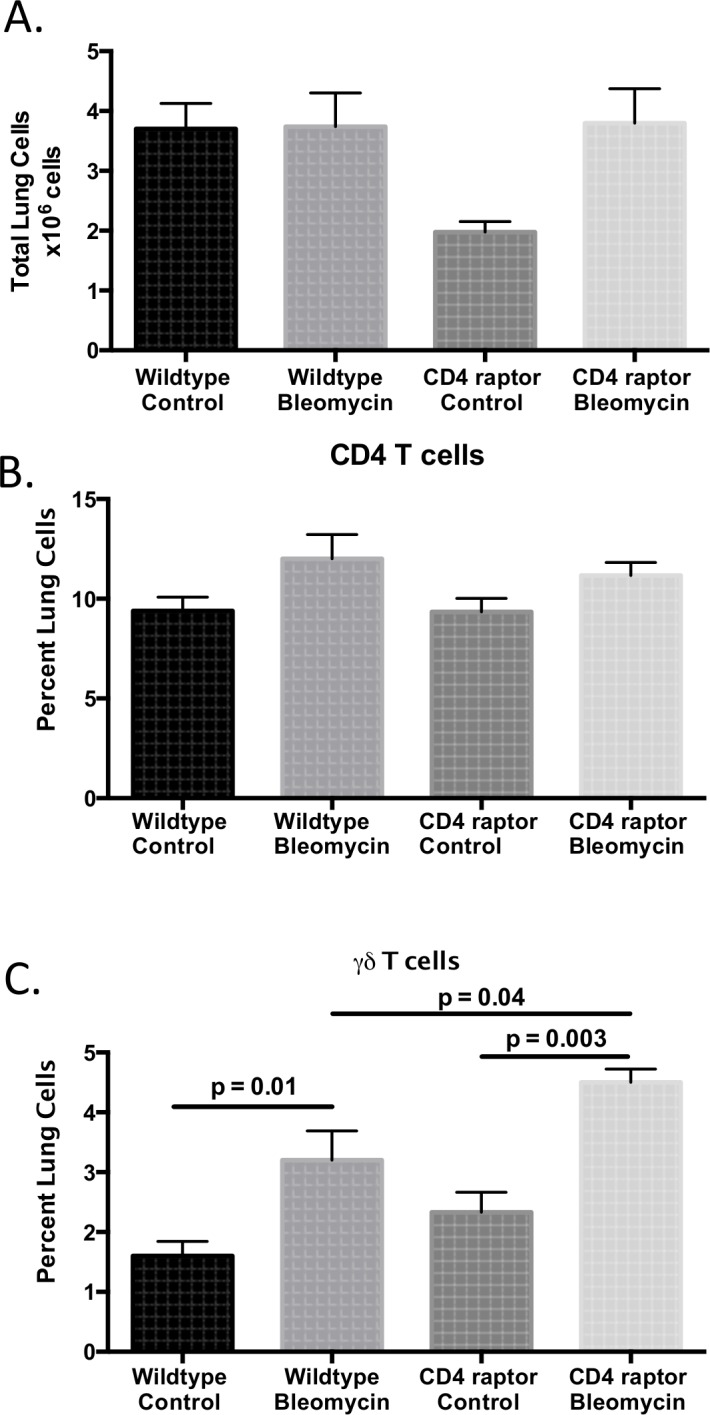
CD4 raptor knockout mice have increased γδ T cells. Day 21 following i.p. bleomycin, lungs were analyzed for (A) total lung cells, (B) percent of CD4+ cells by flow cytometry, (C) percent of γδ T cells by flow cytometry. Data shown from one representative experiment with three replicates, n = 3–6 per group. Error bars represent one standard error. Significance determined by one-way ANOVA followed by Sidak’s multiple comparison’s test.

In order to determine the cause of this difference in mortality and fibrosis, we assessed the inflammatory response within the lungs at day 21. While there was no difference in the total number of lung cells or the percent of CD4+ T cells, there was an increase in the percent of γδ T cells (Fig 2A, 2B and 2C). Both wild type and CD4 raptor knockout mice had an increase in γδ T cells following bleomycin exposure; however, this response was enhanced in the CD4 raptor knockouts.

### Loss of CD4 raptor expression causes a heightened IL-17A+ γδ T cell response

**Fig 3:**
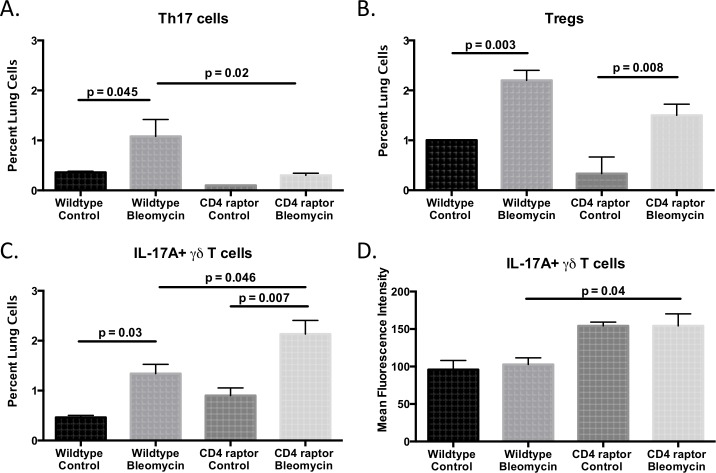
CD4 raptor knockout mice have increased IL-17A+ γδ T cells. Day 21 following i.p. bleomycin, flow cytometric analysis of lung T cells for (A) Th17 cells, (B) regulatory T cells, (C) IL-17A+ γδ T cells. (D) Level of IL-17A expression quantified by mean fluorescence intensity by flow cytometry. Data shown from one representative experiment with three replicates, n = 3–6 per group. Error bars represent one standard error. Significance determined by one-way ANOVA followed by Sidak’s multiple comparison’s test.

Next, we sought to determine the phenotype of the CD4+ T cells and γδ T cells within the lungs. Both wild type and CD4 raptor knockout mice had a paucity of Th1 and Th2 cells, and this did not change after bleomycin treatment (S1A and S1B Fig). As expected, the CD4 raptor knockout mice did not develop the Th17 response that is observed in wild type mice in response to bleomycin (Fig 3A). Both groups did show similar increases in the population of regulatory T cells (Fig 3B). In the absence of a Th17 response, the CD4 raptor knockout mice had an enhanced IL-17A+ γδ T cell response (Fig 3C). These γδ T cells also exhibited heightened production of IL-17A as measured by mean fluorescent intensity of IL-17A expression by flow cytometry (Fig 3D).

### Enhanced IL-17A+ γδ T cell response is associated with increased inflammation and M2 macrophages

**Fig 4:**
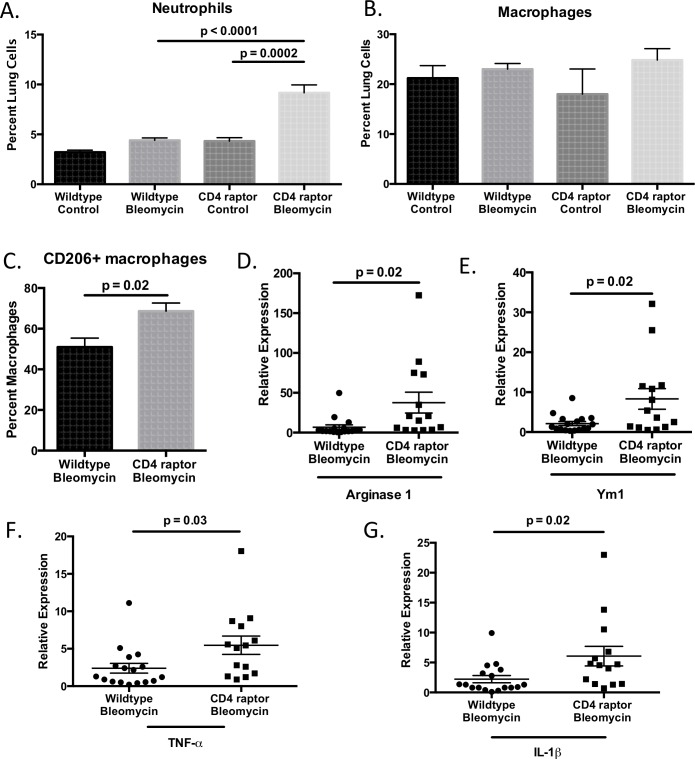
CD4 raptor knockout mice have increased inflammation and M2 macrophages. Day 21 following i.p. bleomycin, lungs were analyzed by flow cytometry for (A) neutrophils, (B) macrophages, (C) CD206+ macrophages. RT-PCR was performed on lungs on day 21 to quantify expression of (D) Arginase 1, (E) YM1, (F) TNF-α, and (G) IL-1β, (A-C) Data shown from one representative experiment with three replicates, n = 3–6 per group. (D-F) Data shown from three pooled experiments, n = 14–17 per group. Error bars represent one standard error. Significance determined by t-test or one-way ANOVA followed by Sidak’s multiple comparison’s test.

In addition to increasing mortality and fibrosis, this enhanced IL-17A+ γδ T cell response seen in the CD4 raptor knockout mice was associated with an enhanced inflammatory response. These mice had more neutrophils within the lungs as well as increased expression of the pro-inflammatory cytokines TNF-α and IL-1β (Fig 4A, 4F and 4G). While there was no difference in the percentage of macrophages, there was a shift in macrophage phenotype, with an increase in the expression of M2 markers Arginase 1 and YM1, as well as an increased percentage of macrophages expressing the M2 marker CD206 (Fig 4B, 4C, 4D and 4E).

## Discussion

Our study reveals the key role of IL-17A+ γδ T cells in promoting the development of pulmonary fibrosis. Prior studies have found that both Th17 cells and γδ T cells are upregulated in animal models of pulmonary fibrosis and produce the cytokine IL-17A. In the absence of IL-17A, either via administration of a neutralizing antibody or use of IL-17A deficient animals, mice develop less fibrosis after bleomycin exposure[7, 9]. Therapies that reduce the population of IL-17A producing cells are similarly able to abrogate and even reverse fibrosis[8, 12, 20]. In order to clarify the roles of Th17 cells and IL-17A+ γδ T cells in this model of pulmonary fibrosis, we utilized a cell-type specific deletion of the mTOR signaling protein raptor to selectively block generation of Th17 cells.

For this study, we utilized a more chronic model of pulmonary fibrosis by administering repeated intraperitoneal injections of bleomycin. We chose this approach because when bleomcyin is administered via a single intratracheal dose, it induces acute inflammation, which progresses to fibrosis[17]. Prior work has shown that the acute and chronic inflammatory responses in bleomycin injury show differential mechanisms of regulation[18]. Thus, in this study, we sought to focus on the chronic inflammation and fibrosis that characterizes human disease.

Initially, we hypothesized that the CD4 raptor knockout mice would have decreased fibrosis. Indeed our data revealed quite the opposite. The increase in pathology seen in the CD4 raptor knockout mice was associated with a marked increase in IL-17A+ γδ T cells in the lungs, thus implicating these cells in promoting inflammation and fibrosis. While Th17 cells also produce the pro-fibrotic cytokine IL-17A, our data indicates that these cells are not necessary for generation of pulmonary fibrosis in this model. Furthermore, our data point out the complexity of responses even when using tissue specific Cre’s. If we had not examined the lungs for γδ T cells, we might have overlooked their contribution with regard to IL-17A production and promotion of disease in this model.

The precise role of γδ T cells in lung fibrosis has been somewhat confusing. One study found that mice overexpressing the transcription factor Egr3 have a higher expression of γδ T cells, and that these mice developed excessive bleomycin-induced lung injury in the setting of an exaggerated response of both Th17 cells and IL-17A+ γδ T cells[11]. Other studies have found that loss of γδ T cells enhances lung fibrosis, through loss of the anti-fibrotic mediators CXCL10, IL-22, and IFN-γ[10, 13–15]. Thus, previous models have purported that Th17 cells promote fibrosis, while γδ T cells have anti-fibrotic effects. Furthermore, studying the role of γδ T cells has been challenging because the antibodies that are used to deplete γδ T cells in fact do not deplete these cells but rather lead to internalization of the γδ T cell receptor, masking them from identification[21]. Our data clarify the pathologic role of γδ T cell production of IL-17A. Additionally, our data indicates that IL-17A+ γδ T cells alone are sufficient to cause pulmonary fibrosis in the absence of Th17 cells. Furthermore, we saw no protective role for these cells despite the absence of Th17 cells. Of note, there was no change in the expression of the cytokines CXCL10, IL-22, and IFN-γ, through which γδ T cells are believed to exert their anti-fibrotic effects (S2A, S2B and S2C Fig).

Mechanistically, while the CD4 raptor knockout mice had an enhanced IL-17A+ γδ T cell response, when balanced with the loss of Th17 cells, there was no change in the total expression of IL-17A within the lungs by PCR and by ELISA (S2D and S2E Fig). However, there was an increase in the expression of TNF-α and IL-1β, as well as an increased neutrophil infiltration of the lungs, and increased M2 macrophage polarization. Taken together, we hypothesize that the increased mortality and fibrosis in the CD4 raptor knockout mice was not due solely to expression of IL-17A, but rather was due to a shift in the inflammatory response, via neutrophil recruitment, M2 macrophage polarization, and upregulation of pro-inflammatory cytokines. Other models of lung injury have similarly found that γδ T cells play a critical role in promoting neutrophil recruitment and M2 macrophage polarization[22–25]. While these effects of γδ T cells may be beneficial to respond to an acute insult such as a bacterial pneumonia, in our chronic model of injury, we hypothesize that it is this persistent neutrophil recruitment, enhanced production of cytokines with inflammatory and pro-fibrotic effects, and M2 macrophage polarization that perpetuates the chronic inflammation and fibrosis.

Prior work has highlighted the role of IL-1β, neutrophils, and M2 macrophages in promoting fibrosis[12, 26–28]. IL-1β is an important pro-fibrotic cytokine, and direct instillation of IL-1β intratracheally has been shown to induce pulmonary inflammation and fibrosis[26]. This has been hypothesized to be due to its effects on promoting IL-17A production both by γδ T cells and Th17 cells[29]. Our study indicates, however, that in the absence of Th17 cells, IL-1β is still able to promote inflammation and fibrosis. Further, the enhanced expression of IL-1β in the absence of Th17 cells may indicate that Th17 cells act as a negative regulator on IL-1β expression. Regarding neutrophils, in patients with IPF, an increase in BAL neutrophils has been found to portend a worse prognosis[30]. Mechanistically, neutrophil elastase has been shown to promote fibroblast and myofibroblast proliferation[28]. M2 macrophages have also been shown to promote pulmonary fibrosis, and a reduction in M2 polarization results in less injury [12, 27].

Our work reveals the important role that IL-17A+ γδ T cells play in promoting the development of pulmonary fibrosis. Th17 cells have been identified as a potential target to treat pulmonary fibrosis, and in animal models, therapies that diminish the Th17 response have been shown to reduce or reverse fibrosis[8, 12]. This data highlights the importance of targeting both Th17 cells and IL-17A+ γδ T cells within the lungs to successful treat pulmonary fibrosis. Additionally, it reveals a novel target for therapy in these IL-17A+ γδ T cells. Targeting these γδ T cells specifically and altering their cytokine production away from IL-17A and towards a phenotype that has been shown to be protective could represent a novel therapeutic avenue.

## Materials and Methods

### Mice

*CD4 Cre* mice and *RAPTOR*^*fl/fl*^ mice on the C57BL/6 background were purchased from The Jackson Laboratory (Bar Harbor, ME), and bred to homozygosity. Mice were housed in a Johns Hopkins University School of Medicine animal facility, kept at 72°F and 45% humidity, with 14 hour dark and 10 hour light exposure. Food was purchased from HARLAN (product number 20185x). Mice had free access to food and water. Eight-to 10-week old female mice were utilized. Mice were euthanized via injection of 100 μl of pentobarbital 20 mg/ml. All animal protocols were approved by the Institutional Animal Care and Use Committee at Johns Hopkins University (Baltimore, MD).

### Reagents

Bleomycin was purchased from App Pharmaceuticals (Schaumbrg, IL). Collagenase type I was purchased from gibo (Waltham, MA). DNase I was purchased from Roche diagnostics (Indianapolis, IN). PMA and Ionomycin were purchased from Sigma-Aldrich (St. Louis, MO). Flow cytometry reagents were purchased from eBioscience (San Diego, CA). Antibodies utilized were from eBioscience: Foxp3 FITC (clone FJK-16s), IL-17A PE (clone eBio17B7), Gr-1 PE (clone RB6-8C5), F4/80 PerCP-Cyanine5.5 (clone BM8), CD8a PerCP-Cyanine5.5 (clone 53–6.7), CD11b PerCP-Cyanine5.5 (clone M1/70), γδ TCR APC (clone eBIOGL3), CD11b APC (clone M1/70); from BioLegend (San Diego, CA): CD4 FITC (clone GK1.5), CD206 FITC (clone C068C2); and from BD Pharmingen (San Jose, CA): IFN-γ PE (clone XMG1.2), CD11c PE (clone HL3), IL-4 PE (clone 11B11), CD4 PerCP (clone RM4-5), CD45 PerCP-Cy5.5 (clone 30-F11). Collagen 1 biotin antibody was purchased from Rockland (Limerick, PA). Strepavidin FITC was purchased from BioLegend (catalog 405202). ELISA IL-17A kit was purchased from eBioscience (San Diego, CA).

### Pulmonary fibrosis model

As previously described, bleomycin was given via intraperitoneal injection (0.64 units per injection in 400 μl PBS) on days 0, 3, 7, 10, 14, 21, and 28 to induce pulmonary fibrosis[12]. Control mice received an equivalent volume of PBS on each day. Mice were harvested on day 21 or day 42. The left lung was taken for lung ELISA, or lung PCR, or histology. The right lung was taken for FACS analysis.

### Histology

Lungs were inflated with formalin to atmospheric pressure 30 cm and sectioned and stained for H&E staining and Masson’s trichrome staining. H&E staining was performed using the Thermo Scientific Richard-Allen Scientific Histology Signature Series Stains and performed according to manufacturer’s instructions. Masson’s trichrome staining was performed using the American MasterTech Masson Trichrome Stain Kit per the manufacturer’s instructions. Images were collected at x20 and x100 magnification using a Nikon Eclipse 80i microscope and Nikon DS-fi1 camera.

### Flow cytometry

Lungs were harvested, chopped with a straight razor, and digested in a solution of collagenase type I (3 mg/ml) and DNase I (200 μl/10 ml) in RPMI media for 60 minutes at 37 C. The homogenates were then filtered through a 70 μm cell strainer to produce a single-cell suspension. Cells were stimulated with PMA (50 ng/ml) and Ionomycin (500 nM) *in vitro* for 3 hours and then stained with the appropriate antibody for FACS analysis. eBioscience fixation/permeabilization reagents were used. Cells were then analyzed with a BD FacsCaliber, BD Biosciences (San Jose, CA), and analyzed using FlowJo software (Ashland, OR).

### RNA extraction and quantitative real time PCR

Lungs were homozenized using an OMNI TH tissue homogenizer (OMNI International, Kennesaw, GA) in Trizol reagent, Invitrogen (Carlsbad, CA), then RNA was extracted using chloroform, precipitated with isopropanol, and finally washed with ethanol to isolate the RNA. RNA was quantified using the NanoDrop 1000 Spectrophotometer (ThermoFisher Scientific, Waltham, MA). cDNA was produced using the BIO-RAD (Hercules, CA) iScript cDNA Synthesis kit per manufacturer’s instructions using a Labnet Multigene Gradient PCR Thermal Cycler (Sigma-Aldrich, St. Louis, MO). Real time PCR was performed on a 7500 Applied Biosystems (Carlsbad, CA) and normalized to 18s RNA. Reagents used for Real Time PCR were purchased from Applied Biosystems. PCR primers were purchased from ThermoFisher Scientific (Waltham, MA): Arg1 (catalogue Mm00475988_m1), Col1a1 (catalogue Mm00801666_g1), CXCL10 (catalogue Mm00445235_m1), IFN-γ (catalogue Mm00801778_m1), IL-1β (catalogue Mm00434228_m1), IL-17A (catalogue Mm00439619_m1), IL-22 (catalogue Mm01226722_g1), TNF-α (catalogue Mm00443258_m1), YM1 (catalogue Mm00657889_mH).

### Whole lung ELISA

Lungs were homogenized in RIPA buffer using an OMNI TH tissue homogenizer (OMNI International, Kennesaw, GA). The total amount of protein was quantified via Bio Rad (Hercules, CA) Quick Start Bradford Assay, and 500 μg of protein was diluted to a volume of 100 μl of ELISA buffer and plated. ELISA was performed using the eBioscience (San Diego, CA) kit per manufacturer’s instructions and analyzed on a Bio-Rad microplate reader model 680.

### Statistical analysis

Statistical analysis was performed using Prism software (GraphPad Software), using a Log-rank test to analyze mortality differences, an unpaired t-test to compare means between two groups, and a one-way ANOVA with a Sidak correction for multiple comparisons to compare means between more than two groups. Statistical significant values were those where *P*<0.05.

## Supporting Information

S1 Fig No change in Th1 and Th2 cells within the lungs following i.p. bleomycin. Day 21 following i.p. bleomycin, flow cytometric analysis of lung T cells for (A) Th1 cells, (B) Th2 cells. Data shown from one representative experiment with three replicates, n = 3–6 per group. Error bars represent one standard error. Significance determined by one-way ANOVA followed by Sidak’s multiple comparison’s test.
(TIF)

S2 Fig No change in lung expression of CXCL10, IL-22, IFN-γ, or IL-17A in CD4 raptor knockout mice. Day 21 following i.p. bleomycin, RT-PCR of lung tissue for expression of (A) CXCL10, (B) IL-22, (C) IFN-γ, and (D) IL-17A. (E) Day 21 following i.p. bleomycin, lung homogenates were analyzed by ELISA to quantify IL-17A. (A-D) Data shown from three pooled experiments, n = 14–17 per group. (E) Data shown from one representative experiment with three replicates, n = 3–6 per group. Error bars represent one standard error. Significance determined by unpaired t-test.
(TIF)
