# Supplementary figures and images for: Deletion of mTORC1 Activity in CD4+ T Cells Is Associated with Lung Fibrosis and Increased γδ T Cells
Source: PLoS One. 2016 Sep 20;11(9):e0163288. doi: 10.1371/journal.pone.0163288 (PMC5029914; doi:10.1371/journal.pone.0163288)

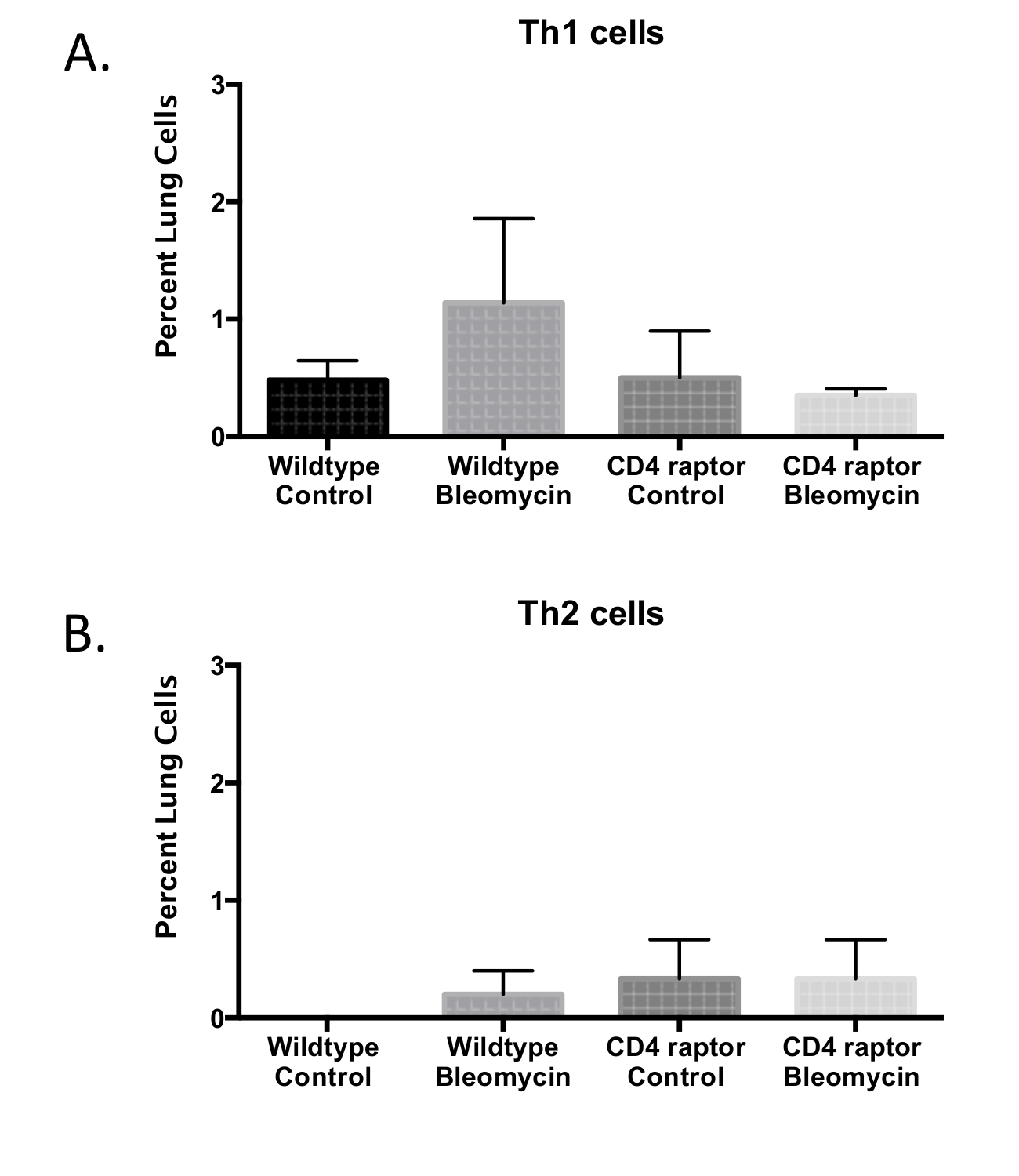

Supplement: S1 Fig — Day 21 following i.p. bleomycin, flow cytometric analysis of lung T cells for (A) Th1 cells, (B) Th2 cells. Data shown from one representative experiment with three replicates, n = 3–6 per group. Error bars represent one standard error. Significance determined by one-way ANOVA followed by Sidak’s multiple comparison’s test. (TIF) [file pone.0163288.s001.tif]

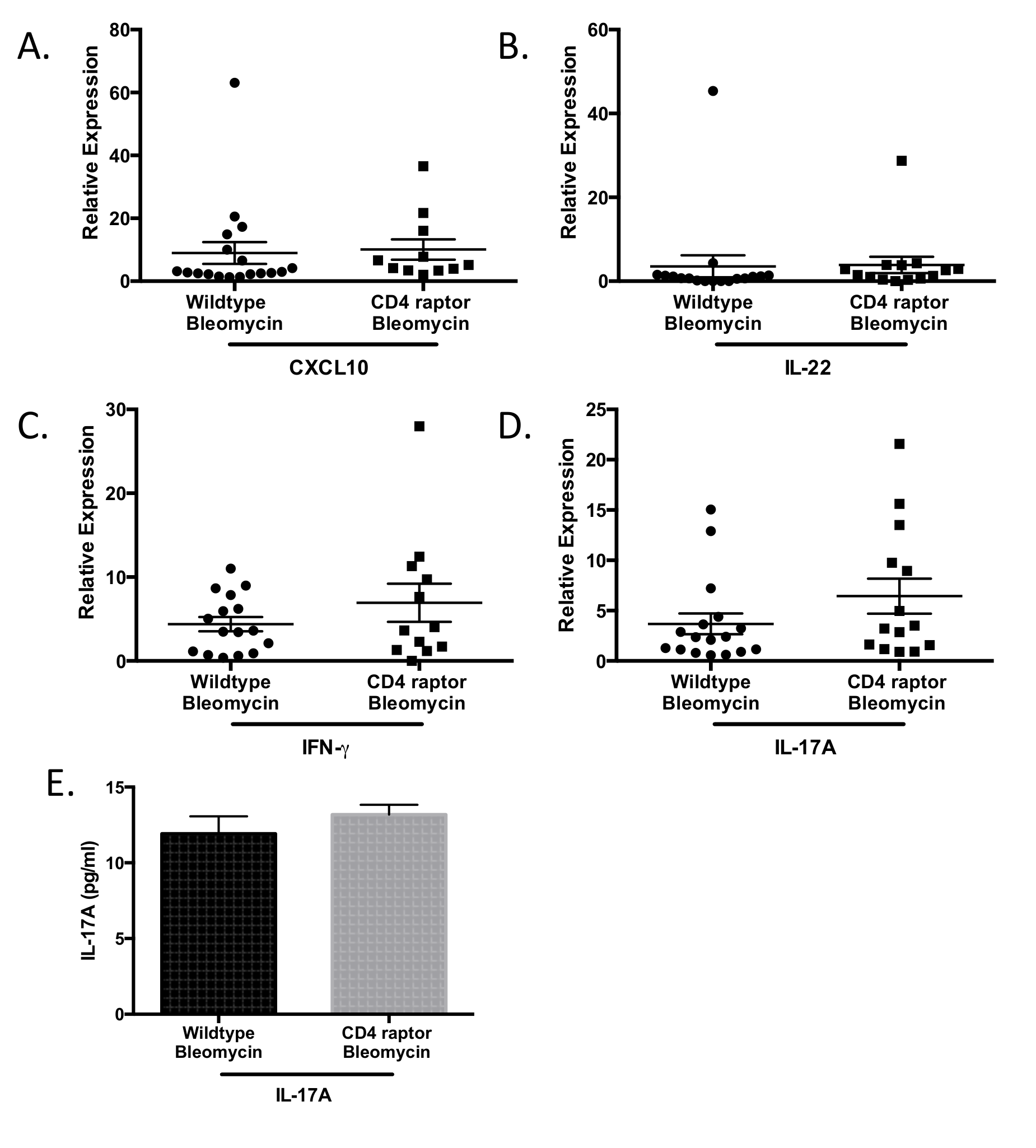

Supplement: S2 Fig — Day 21 following i.p. bleomycin, RT-PCR of lung tissue for expression of (A) CXCL10, (B) IL-22, (C) IFN-γ, and (D) IL-17A. (E) Day 21 following i.p. bleomycin, lung homogenates were analyzed by ELISA to quantify IL-17A. (A-D) Data shown from three pooled experiments, n = 14–17 per group. (E) Data shown from one representative experiment with three replicates, n = 3–6 per group. Error bars represent one standard error. Significance determined by unpaired t-test. (TIF) [file pone.0163288.s002.tif]
